# Canadian Anatomic Kidney Score: Quantitative Macroscopic Assessment of Donor Kidney Quality for Transplantation

**DOI:** 10.1097/TXD.0000000000001604

**Published:** 2024-03-07

**Authors:** Danny Matti, Juliano Offerni, Pavel S. Roshanov, Jirong Lu, Yanbo Guo, Victoria Lebedeva, Erica Ai Li, Haider Abed, William Luke, Alp Sener, Patrick P. Luke

**Affiliations:** 1 Schulich School of Medicine and Dentistry, Western University, London, ON, Canada.; 2 London Health Sciences Centre, London, ON, Canada.; 3 Maxy Rady College of Medicine, University of Manitoba, Winnipeg, MB, Canada.; 4 Division of Urology, McMaster University, Hamilton, ON, Canada.

## Abstract

**Background.:**

The Canadian Anatomic Kidney Score (CAKS) is a novel 6-point grading system that standardizes the gross description of a donor kidney across 3 components—vessels, anatomy, and sticky fat. We hypothesized that the CAKS predicts allograft functional outcomes and provides additional information to the Kidney Donor Profile Index (KDPI) and histologic assessment of the donor kidney.

**Methods.:**

Single-center cohort of 145 patients who underwent renal transplantation with CAKS analysis between 2018 and 2021. CAKS was prospectively determined before transplantation. Preimplantation core biopsies were assessed according to the Remuzzi score (RS). The primary outcome was 1-y allograft function represented by an estimated glomerular filtration rate (eGFR).

**Results.:**

Linear regression without adjustment for KDPI or RS showed a significant association between the CAKS and 1-y eGFR (−8.7 mL/min/1.73 m^2^ per point increase in CAKS; 95% CI, −13.0 to −4.4; *P* < 0.001). Most of that association was attributed to the vessel component (−12.1; −19.4 to −4.8; *P* = 0.002). Adjustment for KDPI and RS attenuated the relationship between 1-y function and CAKS (−4.6; −9.5 to 0.3; *P* = 0.065) and vessel component (−7.4; −15.2 to 0.5; *P* = 0.068).

**Conclusions.:**

Anatomic assessment of donor kidneys at the time of transplantation associates with allograft function at 1 y. Vascular assessment appears to make the dominant contribution.

Decisions to accept or reject a deceased donor kidney often require educated guesses about future graft function. Clinicians increasingly rely on the Kidney Donor Profile Index (KDPI) to inform these guesses. This system, scored 0%–100%, represents percentiles of the Kidney Donor Risk Index (KDRI)—a score that reflects the relative risk of graft failure for deceased donor kidneys. A KDPI of 70% reflects a KDRI >70% (ie, worse) of diseased donor kidneys recovered for transplantation in the United States in the previous calendar year. The KDPI has modest ability to predict allograft outcomes, with particular limitations in the context of acute kidney injury, prolonged diabetes, smoking, or other chronic disease.^1^ To circumvent this, some programs rely on preoperative donor renal biopsies to aid decision-making, but they too have important limitations.

Physical characteristics of the donor kidney, including the presence of atheroma in the donor aorta, presence of cysts, and renal scars are identifiable at the time of organ procurement and can be quantified within seconds. However, a validated quantitative score of macroscopic features of donor kidneys has never been studied or associated with functional allograft outcomes. Therefore, we designed a standardized Canadian Anatomic Kidney Score (CAKS) based on the gross examination of the aortic vasculature, sticky fat around the kidney, and anatomic anomalies of the donor kidney. We hypothesized that a quantitative score of gross donor renal anatomy could predict or serve as an additional tool to help predict functional outcomes of kidney transplants.

## MATERIALS AND METHODS

We report this study in accordance with the Reporting of Observational Studies in Epidemiology Statement.

### Study Procedures

Ethics approval was obtained from the institutional review board (research ethics board: 120254). This was a cohort study of kidney transplants performed at a single Canadian academic hospital from January 1, 2018, to June 30, 2021. Donor characteristics were obtained from the Trillium Gift of Life Network in Ontario, Canada. Recipient characteristics and kidney function outcome data were abstracted from the electronic medical records at our center.

One kidney transplant fellow and an attending transplant surgeon prospectively assigned CAKS to each kidney, which occurred after back-table preparation of the kidney and time zero of transplant. The final score was obtained through their mutual agreement. Determination of the CAKS was completed before implantation and dictated in the operative report according to criteria listed in Table 1. Further, a baseline kidney biopsy was obtained from all kidneys in our center and history was assessed by renal pathologists using the Remuzzi score (RS) criteria.^2^ Neither the CAKS nor the biopsy results informed decisions about whether to proceed with the transplantation. Vascular anomalies such as multiple arteries, arterial interposition, and replacement vessels were not considered. Primary or secondary injuries to the kidney at the time of procurement were not accounted for.

**TABLE 1.** The CAKS component definitions and scoring criteria

Score	Vasculature^*a*^	Sticky fat	Anatomy
	Definition: arteriopathy	Definition: adipose tissue adhered to the renal capsule to an extent that imposes difficulty for removal without damaging the renal capsule	Definition: every additional finding, such as cysts or significant anomalies reported
0	No calcifications in the renal artery or aorta	No sticky fat remains after dissection	No cysts
0.5	Few calcified plaques in the aorta	Almost all sticky fat removed	<3 cysts measuring <3 mm
1.0	Presence of significant calcified plaques or ulcerated plaques in the aorta, but the renal artery is clear	Sticky fat was removed, but there remain areas of intense fibrosis	Between 3 and 6 cysts <3 mm or 1 cyst measuring 3 mm to <1 cm
1.5	When there is a calcified plaque on the ostium of the renal artery	Majority of the fat was intensively adhered and cannot be removed without damaging or opening the renal capsule	Between 7 and 9 cysts or a cyst up to 1–2 cm
2.0	Calcified plaque in the renal artery or any branch	Intense adherent aspect of the renal capsule on top of the intense fat adhesion, which is rendered impossible to remove	>9 cysts or a cyst >2 cm

The Canadian Anatomic Kidney Score (CAKS) is scored of 6 points and is defined by the vasculature, anatomy, and sticky fat content of each kidney. Scores were assigned during the back-table dissection before implantation.

^*a*^Angiodysplasia: The presence of angiodysplasia or aneurysm should add a score of 0.5 in addition to your vasculature score (with a maximum score of 2.0).

The primary study outcome was the recipient’s estimated glomerular filtration rate (eGFR) in mL/min/1.73 m^2^ based on the 2021 CKD-EPI (Chronic Kidney Disease Epidemiology Collaboration equation using a single serum creatinine value collected during routine clinical visits approximately 1 y after transplantation.^2^ The secondary outcome was the presence of eGFR <45 mL/min/1.73 m^2^ (indicating stage 3B chronic kidney disease or worse) at 1 y after transplantation. The surgeons assigned a score to each kidney using Table 1, based on vasculature, anatomy, and sticky fat content of each kidney. Scores were assigned during the back-table dissection before implantation.

All patients were followed according to the standard postoperative protocols established by our multidisciplinary team. They received immunosuppressive therapy, consisting of induction therapy with methylprednisolone and either basiliximab or anti-thymocyte globulin, as well as maintenance therapy with prednisone, mycophenolic acid, and tacrolimus.

### Statistical Analysis

A statistical analysis plan was written before undertaking the analyses. For the primary analyses, we fit linear regression models with the eGFR at 1 y as the dependent variable. The key independent variable was the CAKS, modeled with and without adjustment for the KDPI and RSs. This was then repeated using the individual CAKS and RS components. In the secondary analyses, logistic regression models were used to predict the odds of eGFR <45 mL/min/1.73 m^2^ at 1 y based on the CAKS and, separately, its individual features. These models were also adjusted for KDPI and the RS.

To assess the ability of the CAKS to differentiate between patients who ultimately developed eGFR <45 versus ≥45 mL/min/1.73 m^2^ at 1 y, we plotted receiver operating characteristics curves and calculated the area under the curves with corresponding 95% confidence intervals (CIs). For comparison, we did the same with the KDPI and RS, and with the individual CAKS components.

A tertiary analysis determined if adjusting for the donor’s smoking history might substantially attenuate any association between 1-y graft function and the CAKS, KDPI, and RS.

To handle missing serum creatinine data at 1 y for the 4 patients who died before the 1-y mark after transplantation, the 1-y eGFR was imputed using the most recent preceding creatinine measurement recorded at posttransplant day 7, 3 mo, or 6 mo. Cause of death for 3 of 4 patients was due to unrelated causes and the cause of death for the fourth patient was not specified (Table 2). The cause of death for these patients, along with their corresponding CAKS, were: spontaneous intracranial bleeding, 4; drowning, 0; unknown, 2; hypoxia, 1. For patients known to be receiving dialysis at the time of the most recent measurement, an eGFR value of 5 mL/min/1.73 m^2^ was imputed. In cases of primary nonfunction, an eGFR value of 5 mL/min/1.73 m^2^ was imputed at the 1-y time point. For the 2 patients who did not have serum creatinine values available at the 1-y time point, we imputed the 1-y eGFR using the most recent preceding creatinine measurement at posttransplant day 7, 3 mo, or 6 mo.

**TABLE 2.** Characteristics of patients who died

Characteristics	Summary
	N = 4
Transplant type, n (%)	
Donation after circulatory death	1 (25.0)
Neurologic determination of death	3 (75.0)
KDPI, mean (95% CI)	64.5 (41.9-87.1)
KDPI, n (%)	
<20	0 (0.0)
20–39	0 (0.0)
40–59	2 (50.0)
60–79	1 (25.0)
≥80	1 (25.0)
CAKS total score, median (IQR)	1.5 (0.8–2.5)
CAKS total score, n (%)	
0	1 (25.0)
0.5	0 (0.0)
1	1 (25.0)
1.5	0 (0.0)
2	1 (25.0)
2.5	0 (0.0)
3	0 (0.0)
3.5	0 (0.0)
4	1 (25.0)
CAKS: vessel, n (%)	
0	1 (25.0)
0.5	0 (0.0)
1	2 (50.0)
1.5	0 (0.0)
2	1 (25.0)
CAKS: anatomy, n (%)	
0	4 (100.0)
0.5	0 (0.0)
1	0 (0.0)
1.5	0 (0.0)
2	0 (0.0)
CAKS: sticky fat, n (%)	
0	2 (50.0)
0.5	0 (0.0)
1	1 (25.0)
1.5	0 (0.0)
2	1 (25.0)
Remuzzi total score, median (IQR)	1.0 (0.8–1.3)
Remuzzi total score, n (%)	
0	1 (25.0)
1	2 (50.0)
2	1 (25.0)
3	0 (0.0)
4	0 (0.0)
Remuzzi: glomerular, n (%)	
0	3 (75.0)
1	0 (0.0)
2	1 (25.0)
3	0 (0.0)
Remuzzi: vascular, n (%)	
0	2 (50.0)
1	2 (50.0)
Remuzzi: tubular, n (%)	
0	4 (100.0)
1	0 (0.0)
2	0 (0.0)
Remuzzi: interstitial fibrosis, n (%)	
0	4 (100.0)
1	0 (0.0)
2	0 (0.0)
Early function, n (%)	
IGF	2 (50.0)
SGF	0 (0.0)
DGF	1 (25.0)
Primary nonfunction	1 (25.0)
Serum creatinine (mg/dL) at 1 y, n (%)	
<1	0 (0.0)
1–1.49	1 (25.0)
1.5–1.99	2 (50.0)
≥2	1 (25.0)
Estimated glomerular filtration rate (mL/min/1.73 m^2^) at 1 y, n (%)	
<15 or dialysis	1 (25.0)
15–29	1 (25.0)
30–44	1 (25.0)
45–59	1 (25.0)
60–89	0 (0.0)
≥90	0 (0.0)

CAKS, Canadian Anatomic Kidney Score; CI, confidence interval; DGF, delayed graft function; IGF, immediate graft function; IQR, interquartile range; KDPI, Kidney Donor Profile Index; SGF, slow graft function.

## RESULTS

We initially screened 289 patients but excluded 41 because they did not have a recorded CAKS. We then excluded another 71 patients due to missing RS or KDPI. Finally, we excluded 32 patients because they underwent living donor transplants. Table 3 summarizes the donor characteristics and outcomes for the 145 patients included in this study. Approximately 44% underwent donation after circulatory death transplants, and 55% underwent neurologic determination of death transplants. The mean KDPI was 42. The median CAKS was 0.5 (25th percentile, 0.0; 75th percentile, 1.0), and the median RS was 0.0 (0.0, 1.0). Approximately 30% of the recipients had delayed graft function. The mean eGFR at 1 y after transplantation was 55 mL/min/1.73 m^2^.

**TABLE 3.** Patient characteristics

Characteristics	Summary
	N = 145
Transplant type, n (%)	
Donation after circulatory death	65 (44.8)
Neurologic determination of death	80 (55.2)
KDPI, mean (95% CI)	42 (34.3-46.1)
KDPI, n (%)	
<20	24 (16.6)
20–39	47 (32.4)
40–59	40 (27.6)
60–79	19 (13.1)
≥80	15 (10.3)
CAKS total score, median (IQR)	0.5 (0.0–1.0)
CAKS total score, n (%)	
0	69 (47.6)
0.5	12 (8.3)
1	32 (22.1)
1.5	9 (6.2)
2	16 (11.0)
2.5	2 (1.4)
3	3 (2.1)
3.5	1 (0.7)
4	1 (0.7)
CAKS: vessel, n (%)	
0	85 (58.6)
0.5	18 (12.4)
1	37 (25.5)
1.5	0 (0.0)
2	5 (3.5)
CAKS: anatomy, n (%)	
0	123 (84.8)
0.5	11 (7.6)
1	10 (6.9)
1.5	0 (0.0)
2	1 (0.7)
CAKS: sticky fat, n (%)	
0	118 (81.4)
0.5	2 (1.4)
1	19 (13.1)
1.5	1 (0.7)
2	5 (3.5)
Remuzzi total score, median (IQR)	0.0 (0.0–1.0)
Remuzzi total score, n (%)	
0	76 (52.4)
1	39 (26.9)
2	19 (13.1)
3	6 (4.1)
4	5 (3.5)
Remuzzi: glomerular, n (%)	
0	97 (66.9)
1	39 (26.9)
2	6 (4.1)
3	3 (2.1)
Remuzzi: vascular, n (%)	
0	112 (77.2)
1	33 (22.8)
Remuzzi: tubular, n (%)	
0	135 (93.1)
1	10 (6.9)
2	0 (0.0)
Remuzzi: interstitial fibrosis, n (%)	
0	131 (90.3)
1	13 (9.0)
2	1 (0.7)
Early function, n (%)	
IGF	75 (51.7)
SGF	23 (15.9)
DGF	44 (30.3)
Primary nonfunction	3 (2.1)
Serum creatinine (mg/dL) at 1 y, n (%)	
<1	29 (20.0)
1–1.49	63 (43.5)
1.5–1.99	29 (20.0)
≥2	24 (16.6)
Estimated glomerular filtration rate (mL/min/1.73 m^2^) at 1 y, n (%)	
<15 or dialysis	9 (6.2)
15–29	10 (6.9)
30–44	20 (13.8)
45–59	39 (26.9)
60–89	56 (38.6)
≥90	11 (7.6)

CAKS, Canadian Anatomic Kidney Score; CI, confidence interval; DGF, delayed graft function; IGF, immediate graft function; IQR, interquartile range; KDPI, Kidney Donor Profile Index; SGF, slow graft function.

**FIGURE 1.**
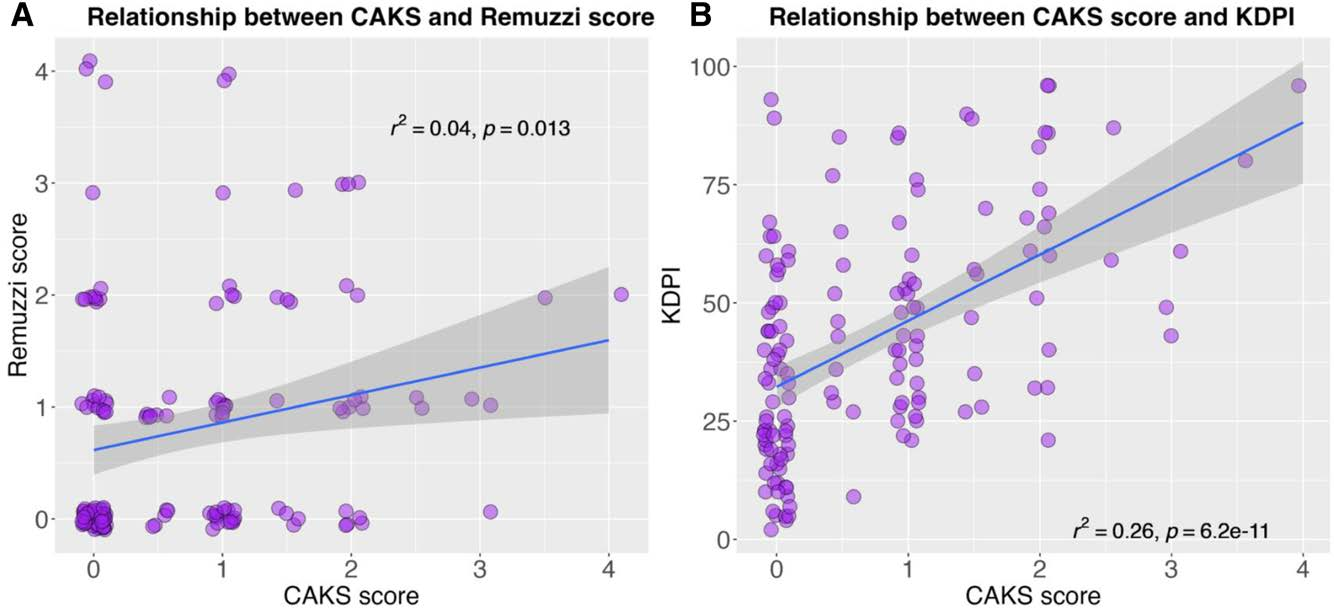
Relationship of Canadian Anatomic Kidney Score (CAKS) with Kidney Donor Profile Index (KDPI) and Remuzzi score. The scatterplots illustrate the relationship between CAKS and the Remuzzi score (A) and the KDPI (B). The *x*-axis in both plots represents the CAKS, whereas the *y*-axis represents the corresponding score being analyzed. The blue regression lines (by the method of least squares) in both plots depict the linear relationships between the CAKS and the respective scores.

There was little correlation between the total CAKS and RS, with variation in 1 explaining only 4% of the variation in the other (Figure 1). CAKS and KDPI were more closely associated: variation in 1 could explain 25% of variation in the other (*P* < 0.001).

**FIGURE 2.**
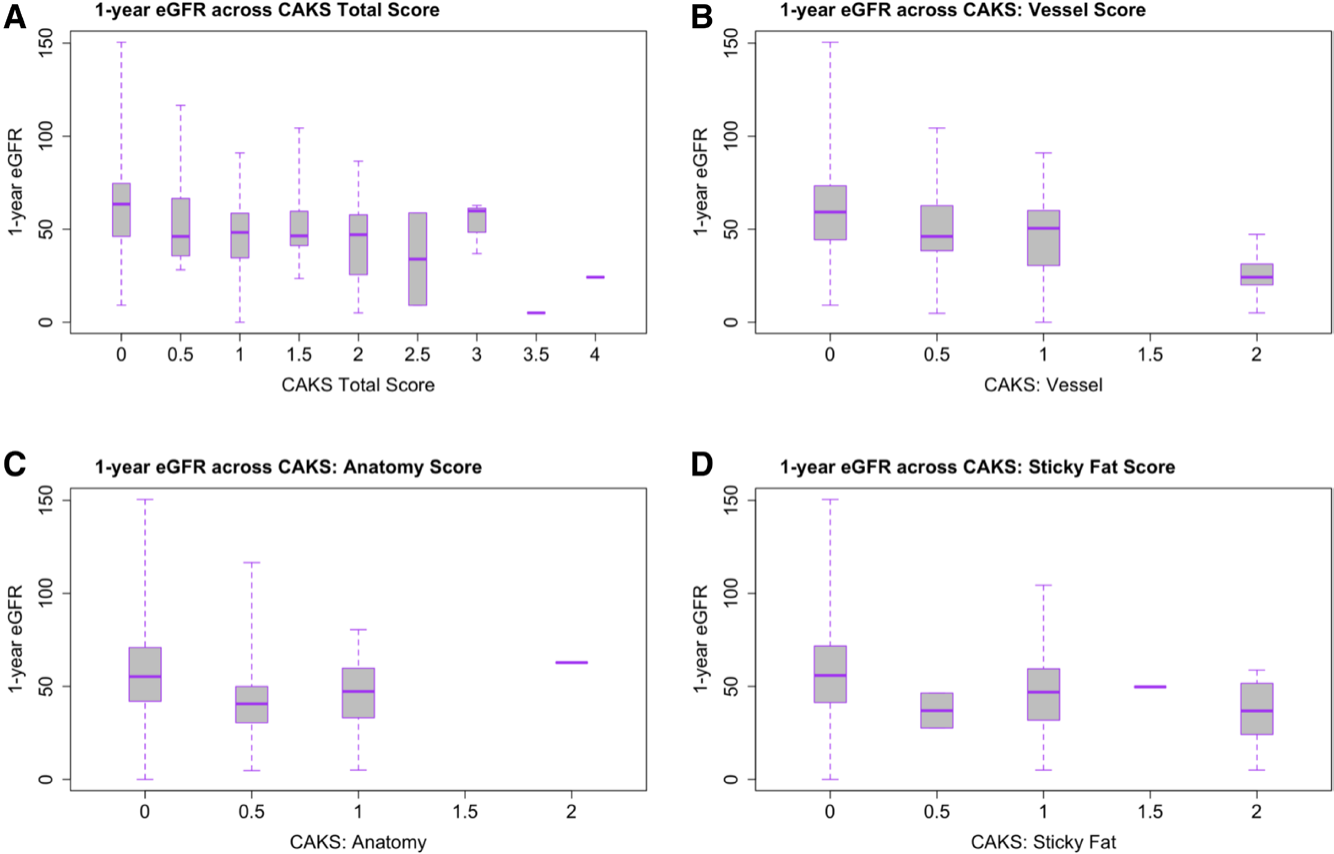
One-year estimated glomerular filtration rate (eGFR) across Canadian Anatomic Kidney Score (CAKS) total score and its components. Boxplots of 1-y eGFR across (A) CAKS total score, (B) CAKS: vessel score, (C) CAKS: anatomy score, and (D) CAKS: sticky fat score. The whiskers represent 95% confidence intervals.

Figure 2 demonstrates 1-y eGFR across categories of the CAKS and its components. Table 4 summarizes the results of the primary analyses with 1-y eGFR as the outcome of interest. Without adjustment for the KDPI or RS, we found significant association between the CAKS and 1-y eGFR (−8.7 mL/min/1.73 m^2^ per point increase in CAKS; 95% CI, −13.0 to −4.4; *P* < 0.001). When score components were analyzed as separate variables in the same model, the vessel component remained strongly associated with 1-y eGFR (−12.1; −19.4 to −4.8; *P* = 0.002) while the anatomy and sticky fat components were poorly associated with eGFR. Adjusting for the KDPI and RS attenuated the relationship between 1-y eGFR and the CAKS (−4.6; −9.5 to 0.3; *P* = 0.065) as well as the vessel component (−7.4; −15.2 to 0.5; *P* = 0.068), whereas the anatomy and sticky fat components continued to provide limited independent predictive information.

**TABLE 4.** Primary analysis: associations of CAKS, KDPI, and Remuzzi score with eGFR at 1 y

Characteristic	CAKS-only model		CAKS + KDPI		CAKS + KDPI + Remuzzi	
	Beta (95% CI)	*P*	Beta (95% CI)	*P*	Beta (95% CI)	*P*
Total scores						
CAKS total score	−8.7 (−13.0 to −4.4)	<0.001	−4.8 (−9.6 to 0.1)	0.056	−4.6 (−9.5 to 0.3)	0.065
KDPI			−0.3 (−0.5 to −0.1)	0.002	−0.3 (−0.5 to −0.1)	0.005
Remuzzi total score					−1.6 (−5.2 to 2.0)	0.386
Individual components						
CAKS: vessel	−12.1 (−19.4 to −4.8)	0.002	−7.6 (−15.3 to 0.2)	0.057	−7.4 (−15.2 to 0.5)	0.068
CAKS: anatomy	−4.7 (−16.5 to 7.1)	0.437	−2.9 (−14.4 to 8.7)	0.627	−3.4 (−15.2 to 8.4)	0.575
CAKS: sticky fat	−6.5 (−14.3 to 1.3)	0.106	−2.6 (−10.6 to 5.4)	0.526	−2.8 (−11.1 to 5.4)	0.499
KDPI			−0.3 (−0.5 to −0.1)	0.003	−0.3 (−0.5 to −0.1)	0.008
Remuzzi: interstitial fibrosis					2.4 (−11.1 to 15.8)	0.731
Remuzzi: tubular					−10.0 (−27.0 to 7.0)	0.250
Remuzzi: vascular					−0.3 (−9.5 to 9.0)	0.954
Remuzzi: glomerular					−1.0 (−7.0 to 5.0)	0.737

Association between CAKS total score and eGFR at 1 y after transplantation estimated in 3 models: a model with only the CAKS total score variable (left), a model adjusting for KDPI (middle), and a model adjusting for KDPI and Remuzzi total score (right). Beta coefficients represent the difference in mean eGFR at 1 y associated with a 1-point increase in the predictor variables (eg, increase from KDPI 30 to KDPI 31, increase in CAKS from 0 to 1, etc).

CAKS, Canadian Anatomic Kidney Score; CI, confidence interval; eGFR, estimated glomerular filtration rate; KDPI, Kidney Donor Profile Index.

Table 5 summarizes the secondary analyses with the occurrence of eGFR <45 mL/min/1.73 m^2^ as the outcome of interest. This outcome occurred in 39 (26.9%) of patients at 1 y after transplant. Without adjusting for the KDPI or RS, a significant association was found between CAKS and the occurrence of eGFR <45 mL/min/1.73 m^2^ 1 y posttransplant (odds ratio [OR] per point increase in CAKS, 1.7; 95% CI, 1.2 to 2.7; *P* = 0.009). When analyzing the score components as separate variables within the same model, the vessel component remained associated with the occurrence of eGFR <45 mL/min/1.73 m^2^ 1-y posttransplant (OR, 2.3; 95% CI, 1.1 to 4.436 *P* = 0.021), whereas the anatomy and sticky fat components were not significantly associated with that outcome. Adjusting for the KDPI and RS attenuated the relationship between the occurrence of eGFR <45 mL/min/1.73 m^2^ 1-y posttransplant and the CAKS (OR, 1.2; 95% CI, 0.7 to 1.9; *P* = 0.478) as well as the vessel component (OR, 1.5; 95% CI, 0.7 to 3..2; *P* = 0.329), whereas the anatomy and sticky fat components continued to provide limited independent predictive information. Adding smoking as a covariate in tertiary analyses did not appreciably change the results (Table S1, **SDC**, http://links.lww.com/TXD/A631 and Table S2, **SDC**, http://links.lww.com/TXD/A631).

**TABLE 5.** Secondary analysis: associations of CAKS, KDPI, and Remuzzi score with occurrence of eGFR < 45 mL/min/1.73 m^2^ at 1 y

Characteristics	CAKS-only model		CAKS + KDPI		CAKS + KDPI + Remuzzi	
	OR (95% CI)	*P*	OR (95% CI)	*P*	OR (95% CI)	*P*
Total scores						
CAKS total score	1.7 (1.2-2.7)	0.009	1.2 (0.7-2.0)	0.441	1.2 (0.7-1.9)	0.478
KDPI			1.0 (1.0-1.0)	0.003	1.0 (1.0-1.0)	0.006
Remuzzi total score					1.2 (0.8-1.8)	0.288
Individual components						
CAKS: vessel	2.3 (1.1-4.6)	0.021	1.5 (0.7-3.3)	0.293	1.5 (0.7-3.2)	0.329
CAKS: anatomy	1.4 (0.4-4.3)	0.537	1.2 (0.4-3.8)	0.712	1.2 (0.3-3.6)	0.797
CAKS: sticky fat	1.4 (0.7-2.9)	0.347	1 (0.4-2.1)	0.898	0.9 (0.4-2.0)	0.725
KDPI			1.0 (1.0-1.1)	0.003	1.0 (1.0-1.0)	0.008
Remuzzi: interstitial fibrosis					0.7 (0.1-2.6)	0.593
Remuzzi: tubular					1.0 (0.1-5.8)	0.970
Remuzzi: vascular					1.8 (0.7-4.6)	0.204
Remuzzi: glomerular					1.2 (0.7-2.2)	0.509

Association between CAKS total score and occurrence of eGFR < 45 mL/min/1.73 m^2^ at 1 y after transplantation estimated in 3 models: a model with only the CAKS total score variable (left), a model adjusting for KDPI (middle), and a model adjusting for KDPI and Remuzzi total score (right). ORs represent the likelihood of eGFR < 45 mL/min/1.73 m^2^ at 1 y associated with a 1-point increase in the predictor variables (eg, increase from KDPI 30 to KDPI 31, increase in CAKS from 0 to 1, etc).

CAKS, Canadian Anatomic Kidney Score; CI, confidence interval; eGFR, estimated glomerular filtration rate; KDPI, Kidney Donor Profile Index; OR, odds ratio.

Table 6 outlines the association between CAKS, KDPI, and RS with the occurrence of dialysis. There is a small number of patients who require dialysis (n = 9). Given this, there was a statistically significant association when considering CAKS total score alone with an OR of 2.4 (95% CI, 1.2-5.0, *P* = 0.016), in combination with KDPI with an OR of 3.7 (95% CI, 1.5-10.4, *P* = 0.008), and when CAKS, KDPI and RS were combined for an OR of 3.6 (95% CI, 1.4-10.2, *P* = 0.009). No statistical significance predictive relationship was achieved when evaluating the individual components of CAKS. This small sample size prevents us from drawing robust conclusions.

**TABLE 6.** Associations of CAKS, KDPI, and Remuzzi score with occurrence of dialysis (eGFR < 15 mL/min/1.73 m^2^) at 1 y

Characteristics	CAKS-only model		CAKS + KDPI		CAKS + KDPI + Remuzzi	
	OR (95% CI)	*P*	OR (95% CI)	*P*	OR (95% CI)	*P*
Total scores						
CAKS total score	2.4 (1.2-5.0)	0.016	3.7 (1.5-10.4)	0.008	3.6 (1.4-10.2)	0.009
KDPI			1.0 (0.9-1.0)	0.171	1.0 (0.9-1.0)	0.127
Remuzzi total score					1.5 (0.7-2.9)	0.261
Individual components						
CAKS: vessel	2.4 (0.6-8.8)	0.203	4.1 (0.8-20.3)	0.077	4.6 (0.9-31.9)	0.091
CAKS: anatomy	2.8 (0.4-13.2)	0.211	3.3 (0.5-16.1)	0.157	3.7 (0.5-20.0)	0.134
CAKS: sticky fat	2.3 (0.7-6.7)	0.157	3.7 (0.9-14.6)	0.055	3.9 (0.9-15.7)	0.053
KDPI			1.0 (0.9-1.0)	0.172	1.0 (0.9-1.0)	0.136
Remuzzi: interstitial fibrosis					1.0 (0.0-15.5)	0.997
Remuzzi: tubular					3.4 (0.1-85.2)	0.470
Remuzzi: vascular					2.3 (0.4-13.2)	0.345
Remuzzi: glomerular					1.0 (0.2-3.4)	0.991

Association between CAKS total score and occurrence of eGFR<15 mL/min/1.73 m^2^ at 1 y after transplantation estimated in 3 models: a model with only the CAKS total score variable (left), a model adjusting for KDPI (middle), and a model adjusting for KDPI and Remuzzi total score (right). Odds ratios represent the likelihood of eGFR<15 mL/min/1.73 m^2^ at 1 y associated with a 1-point increase in the predictor variables (eg, increase from KDPI 30 to KDPI 31, increase in CAKS from 0 to 1, etc).

CI, confidence interval; eGFR, estimated glomerular filtration rate; CAKS, Canadian Anatomic Kidney Score; KDPI, Kidney Donor Profile Index; OR, odds ratio.

**FIGURE 3.**
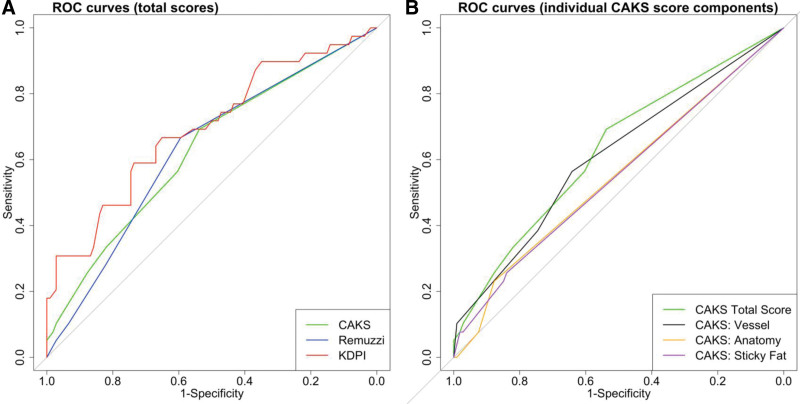
Receiver operating characteristics (ROC) curves comparing scores and their components. ROC curves were drawn from unadjusted models for the outcome estimated glomerular filtration rate (eGFR) <45 mL/min/1.73 m^2^, which occurred in 39 (26.9%) patients at 1 year after transplantation. Area under the curve (AUC) (95% confidence interval [CI]): Canadian Anatomic Kidney Score (CAKS), 0.63 (0.53-0.73); Remuzzi, 0.63 (0.53-0.72); KDPI, 0.69 (0.59-0.80); CAKS: vessel, 0.61 (0.51-0.71); CAKS: anatomy, 0.55 (0.48-0.62); CAKS: sticky fat, 0.55 (0.47-0.63). KDPI, Kidney Donor Profile Index.

Figure 3A compares the ROC curves for the CAKS, RS, and KDPI—an indicator of their ability to discriminate between patients who developed eGFR < 45 and ≥ 45 mL/min/1.73 m^2^ at 1 y. There was no significant difference in discrimination ability of the CAKS compared with either the RS or KDPI. The vessel component had similar discrimination ability to the CAKS total score (Figure 3B).

## DISCUSSION

The CAKS gross anatomic assessment of donor kidneys is associated with 1-y graft function. The association is clear in unadjusted analyses and strongly suggested in analyses adjusted for KDPI and the RS.

The physical attributes of donor kidneys are often not communicated or formally considered during perioperative decision-making even if the donor team identifies significant atherosclerotic disease in the vessels. Unlike a perioperative biopsy, there is no time penalty nor a significant cost in conveying this score to the transplanting team. Additionally, a biopsy at the time of transplantation provides analysis using a limited amount of representative tissue and it has been further shown that biopsies cannot exclude kidneys that are unusable.^3^ Further, the use of biopsy is not routine practice at our center. There may be utility in kidneys with a borderline KDPI score, however sample of these kidneys was limited in our study. Our study aims to look at the role of CAKS as an adjunct to both KDPI and RS. A more heterogeneous population of donor kidneys with varying KDPI and RS can be introduced if a larger, multicenter study is done in the future.

Sticky fat, arteriopathy, and anatomical abnormalities (primarily cysts) were used in the construction of the CAKS. The presence of sticky fat around the renal capsule was investigated by surgeons interested in partial nephrectomy procedures.^4,5^ Most surgeons have anecdotally correlated the presence of this fat to older donors with hypertension or diabetes, but have not evaluated the presence of sticky fat with renal functional outcomes in transplantation. Radiologic evidence of renal cysts has also been shown to be correlated with chronic kidney disease,^6,7^ although the quantitative assessment of the presence of cysts has not been assessed in donor kidney performance, and is rarely described by donor teams. Finally, the presence of atheroma in the aorta and renal artery can be readily seen by the donor surgeon in deceased donors. Although the presence of diseased vessels is believed to be a harbinger of histopathologic arteriosclerotic disease in the kidney, a validated quantified assessment of macrovascular atheroma has not been developed, nor has it been compared with biopsy-proven arterial disease in the kidney. Other anatomic anomalies such as multiple vessels, ureters, and donor-related injury had been considered in creating the CAKS but were excluded as we did not believe they would influence the functional capacity of the donor kidney even if they may affect the decision to use or discard the donor organ.

To our knowledge, this is the first study to examine the performance of a quantitative score based purely upon gross anatomic characteristics in predicting posttransplant renal function. There remains a paucity of literature testing a macroscopic scoring system for organ transplantation, with few currently in existence for renal allografts. A kidney quality score developed by Nicholson examined macroscopic graft appearance, renal blood flow, and urine output after 1 h of normothermic perfusion.^8,9^ Their macroscopic assessment consisted of a perfusion grade based on renal graft color: pink, patchy, mottled, or purple/black. This score was initially assessed in kidneys that were unsuitable for transplantation. In a later series of 36 kidney transplants, grafts with a higher score had a higher incidence of delayed graft function and lower 1-y GFR.^10^ However, this assessment is limited by the availability of normothermic perfusion pumps and the ability to pump away perfusion defects with machine perfusion. There is also research examining the correlation between kidney stiffness detected on ultrasound elastography with histopathologic abnormalities detected on renal biopsy.^11-13^ Although presence of fibrosis was initially considered in our research, the subjective aspect of tactile fibrosis relies on the surgeon’s expertise in elastography and its day-to-day availability. Although we considered adding tactile presence of ‘fibrosis’ in the CAKS, but ultimately determined that its assessment was not reliably reproducible.

Our study has limitations. The kidney grafts at our institution are relatively homogenous and high-quality, with a mean KDPI of 42 and a narrow deviation from this mean; this reduced the number of cases with poor 1-y function in our analysis. In other words, most grafts scored a 0 or 1 for both the CAKS and pathologic total score, with fewer kidneys having a higher score. This limits detection of association between total CAKS across and 1-y kidney function. We are currently studying the reproducibility and ease of use of the CAKS in the hands of trainees and experienced transplant surgeons from programs across Canada.

If the CAKS is to be adopted, we recommend documenting and communicating the separate CAKS component scores because they clearly differ in their association with 1-y graft function. The vascular score predicted graft function best. Sticky fat and anatomic anomalies (mostly cysts), on the other hand, did not independently predict graft function. However, our cohort had few kidneys with cysts or sticky fat. It is possible that with greater numbers of marginal kidneys analyzed, the presence of renal cysts may emerge as a stronger predictor of poor graft function than indicated in this initial analysis. Nevertheless, in our refinement of the CAKS as a clinical prediction guide, the weight of these factors may become downgraded or phased out altogether.

In conclusion, the CAKS is a novel grading system that standardizes the gross description of the donor kidney and associates it with 1-y allograft function. Vascular assessment appears to make the dominant contribution. Our study indicates that CAKS may offer modest additional prognostic information beyond what is provided by KDPI. It is important to emphasize that CAKS is not a replacement for KDPI, rather it has the potential to complement it as a tool by contributing additional insights.

P.S.R. holds a competitive salary award from the Academic Medical Organization of Southwestern Ontario and receives research funding support from the Department of Medicine at Western University and from the William F. Clark endowed chair in Nephrology. The other authors declare no conflicts of interest.

## Supplementary Material


