# Supplementary material for: Canadian Anatomic Kidney Score: Quantitative Macroscopic Assessment of Donor Kidney Quality for Transplantation
Source: Transplant Direct. 2024 Mar 7;10(4):e1604. doi: 10.1097/TXD.0000000000001604 (PMC10923352; doi:10.1097/TXD.0000000000001604)
Supplement: Supplementary file 1 [file txd-10-e1604-s001.pdf]

Table S1. Tertiary analysis: associations of CAKS, smoking status, KDPI, and Remuzzi score with eGFR at 1 year.

	CAKS + Smoking		CAKS + KDPI + Smoking		CAKS + KDPI + Remuzzi + Smoking	
**Characteristic**	**Beta (95% CI)**	**p-value**	**Beta (95% CI)**	**p-value**	**Beta (95% CI)**	**p-value**
**Total Scores**						
CAKS Total Score	-8.9 (-13.1 to -4.7)	<0.001	-4.3 (-9.0 to 0.5)	0.081	-4.3 (-9.1 to 0.5)	0.079
Smoking	-0.7 (-8.2 to 6.8)	0.862	-1.3 (-8.4 to 5.9)	0.731	-1.2 (-8.4 to 6.0)	0.748
KDPI			-0.3 (-0.5 to -0.2)	<0.001	-0.3 (-0.5 to -0.2)	<0.001
Remuzzi Total Score					-0.7 (-3.1 to 4.5)	0.723
**Individual Components**						
CAKS: Vessel	-13.2 (-20.4 to -5.9)	<0.001	-7.8 (-15.4 to -0.3)	0.044	-7.90 (-15.6 to -0.23)	0.046
CAKS: Anatomy	-5.5 (-17.1 to 6.0)	0.350	-3.6 (-14.7 to 7.6)	0.529	-2.88 (-14.3 to 8.51)	0.621
CAKS: Sticky Fat	-5.5 (-13.3 to 2.3)	0.168	-0.7 (-8.6 to 7.2)	0.860	0.32 (-7.9 to 8.6)	0.938
Smoking	-0.4 (-7.9 to 7.1)	0.909	-1.1 (-8.4 to 6.1)	0.756	-1.07 (-8.4 to 6.3)	0.777
KDPI			-0.3 (-0.5 to -0.2)	<0.001	-0.34 (-0.5 to -0.2)	<0.001
Remuzzi: Interstitial Fibrosis					7.50 (-6.2 to 21.2)	0.284
Remuzzi: Tubular					0.58 (-17.3 to 18.5)	0.949
Remuzzi: Vascular					-0.98 (-10.1 to 8.2)	0.833
Remuzzi: Glomerular					-0.39 (-6.3 to 5.5)	0.897

Footnote: Association between CAKS Total Score, smoking status, and eGFR at 1 year after transplantation estimated in three models: a model with the CAKS total score variable and smoking status (left), a model adjusting for KDPI (middle), and a model adjusting for KDPI and Remuzzi Total Score (right). Beta coefficients represent the difference in mean eGFR at 1 year associated with a 1-point increase in the predictor variables (e.g., increase from KDPI 30 to KDPI 31, increase in CAKS from 0 to 1, etc). Abbreviations: CI, confidence interval; CAKS, Canadian Anatomic Kidney Score; KDPI, Kidney Donor Profile Index.

Table S2. Tertiary analysis: associations of CAKS, smoking status, KDPI, and Remuzzi score with eGFR <45 ml/min/$1.73m^2$ at 1 year.

	CAKS + Smoking		CAKS + KDPI + Smoking		CAKS + KDPI + Remuzzi + Smoking	
**Characteristic**	**OR (95% CI)**	**p-value**	**OR (95% CI)**	**p-value**	**OR (95% CI)**	**p-value**
**Total Scores**						
CAKS Total Score	1.8 (1.2 to 2.7)	0.007	1.2 (0.7 to 1.9)	0.547	1.2 (0.7 to 1.9)	0.555
Smoking	1.3 (0.6 to 2.9)	0.513	1.4 (0.6 to 3.2)	0.439	1.4 (0.6 to 3.3)	0.435
KDPI			1.0 (1.0 to 1.1)	<0.001	1.0 (1.0 to 1.1)	0.002
Remuzzi Total Score					1.1 (0.7 to 1.6)	0.751
**Individual Components**						
CAKS: Vessel	2.5 (1.2 to 5.3)	0.011	1.6 (0.7 to 3.6)	0.258	1.7 (0.7 to 3.9)	0.214
CAKS: Anatomy	1.5 (0.5 to 4.7)	0.464	1.3 (0.4 to 4.2)	0.612	1.1 (0.3 to 3.6)	0.850
CAKS: Sticky Fat	1.3 (0.6 to 2.7)	0.475	0.8 (0.3 to 1.8)	0.594	0.6 (0.2 to 1.4)	0.235
Smoking	1.3 (0.6 to 2.9)	0.549	1.4 (0.6 to 3.2)	0.467	1.3 (0.5 to 3.2)	0.553
KDPI			1.0 (1.0 to 1.1)	<0.001	1.0 (1.0 to 1.1)	0.001
Remuzzi: Interstitial Fibrosis					0.4 (0.0 to 1.9)	0.301
Remuzzi: Tubular					0.2 (0.0 to 2.1)	0.224
Remuzzi: Vascular					2.0 (0.7 to 5.3)	0.182
Remuzzi: Glomerular					1.3 (0.7 to 2.4)	0.470

Footnote: Association between CAKS Total Score, smoking status, and occurrence of eGFR<45 ml/min/$1.73m^2$ at 1 year after transplantation estimated in three models: a model with the CAKS total score variable and smoking status (left), a model adjusting for KDPI (middle), and a model adjusting for KDPI and Remuzzi

Total Score (right). Odds ratios represent the likelihood of eGFR<45 ml/min/1.73m^2 at 1 year associated with a 1-point increase in the predictor variables (e.g., increase from KDPI 30 to KDPI 31, increase in CAKS from 0 to 1, etc). Abbreviations: CI, confidence interval; OR, odds ratio; CAKS, Canadian Anatomic Kidney Score; KDPI, Kidney Donor Profile Index.
